# Short stature with precocious puberty caused by aggrecan gene mutation: A case report

**DOI:** 10.1097/MD.0000000000021635

**Published:** 2020-08-21

**Authors:** Yuanyuan Wang, Juan Ge, Jianying Ma, Lingyan Qiao, Tang Li

**Affiliations:** a Qingdao Women and Children's Hospital, Cheeloo College of Medicine, Shandong University; b Department of Pediatrics, Weifang. Maternal and Children Health Hospital, Weifang; c Department of Pediatric endocrinology and metabolism, Qingdao Women and Children's Hospital; d Qingdao Hiser Hospital, Qingdao, China.

**Keywords:** aggrecan gene, central precocious puberty, missense variant, small for gestational age, short stature

## Abstract

**Introduction:**

The present study is carried out to review the clinical data and gene detection results of a pediatric patient with short stature, and to summarize the relationship between clinical phenotype and genotype of the child with Aggrecan *(ACAN)* gene mutation.

**Patient concerns:**

Our study was started with the observation and follow-up of a 5-year-4-month-old full-term child with short stature accompanied by central precocious puberty (CPP).

**Diagnosis:**

Gene sequencing showed that there was a new heterozygous mutation C.2164C >G(p.P722A) in exon 11 of *ACAN* gene, which was inherited from her father.

**Interventions:**

The child was treated by growth hormone for 6 months with mild growth, and accelerated bone age (BA) after the presence of precocious puberty. The child was diagnosed with CPP, and was provided with combined gonadotropinreleasing hormone (GnRH) therapy.

**Outcomes:**

The height of the pediatric patient was 99.4 cm (-3.13SDS) on admission, which was 111.9 cm (-2.08SDS) at the age of 6 years and 10 months, with a growth rate of 8.1 cm/year. There was no significant increase in BA of the pediatric patient after 1 year of follow-up.

**Conclusion:**

Literature review indicated that the clinical manifestations of *ACAN* gene mutation are the most common in idiopathic short stature, most of which are familial inheritance and can also be sporadic. Some children may also have osteoarthritis, disc herniation or degeneration. In most cases, children may have advanced BA, and retardation of BA is also found in some cases. To sum up, growth hormone combined with GnRH analogue treatment can effectively improve body height of children by postponing their adolescence. Meanwhile, *ACAN* gene mutation shall be considered for small-for-gestational-age children without significant growth catch-up and with family history.

## 1 Introduction

Short stature refers to those who are significantly shorter than the average height of the normal population by 2 standard deviations or below the third percentile of normal children in similar living environment of the same region, gender and age.^[1]^ The etiology of short stature is relatively complex, which may be associated with growth hormone (GH) deficiency, idiopathic short stature (ISS), hypothyroidism, malnutrition, constitutional delay of growth and puberty, intrauterine growth retardation, Turner syndrome, cartilage dysplasia, chronic liver and kidney diseases, etc.^[2]^ At present, only a small number of children with short stature can be diagnosed, resulting in a large proportion of children with short stature diagnosed as ISS. With the realization of exome sequential analysis at present, some ISS children have been diagnosed definitely. Besides, increasingly more attention has been paid to the relationship between Aggrecan *(ACAN)* gene mutation (OMIM No. 165800) and short stature. In this study, a case of short stature and idiopathic central precocious puberty (CPP) caused by *ACAN* gene mutation was diagnosed by high-throughput sequencing, and the report is as follows:

## 2 Case presentation

**Figure 1:**
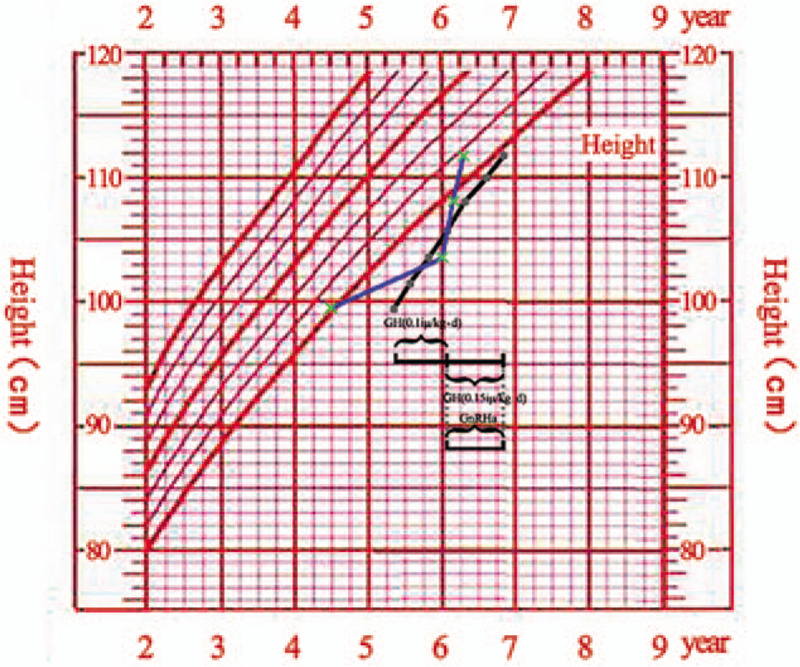
Black curve: the growth curve of the patient with heterozygous ACAN mutation; Blue curve: BA monitoring at the age of 5 years and 4 months, 5 years and 10 months, 6 years and 4 months and 6 years and 10 months respectively; Black line: rhGH (0.1iu/kg.d) treatment in the patient from the age of 5 years and 4 months to 5 years and 10 months, dosage-added rhGH (0.15iu/kg.d) treatment after the age of 5 years and 10 months; and simultaneous add-on therapy of GnRHa. ACAN = aggrecan, GnRHa = gonadotropin releasing hormone analoges.

**Figure 2:**
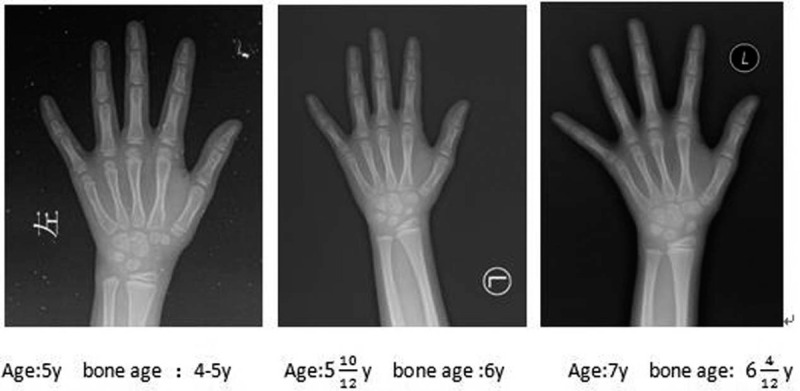
Age corresponds to changes in bone age. During the 6-month period from 5 years and 4 months to 5 years and 10 months, bone age increased rapidly. During the 1-year follow-up, there was no significant increase in bone age from 5 years and 10 months to nearly 7 years.

**Figure 3:**
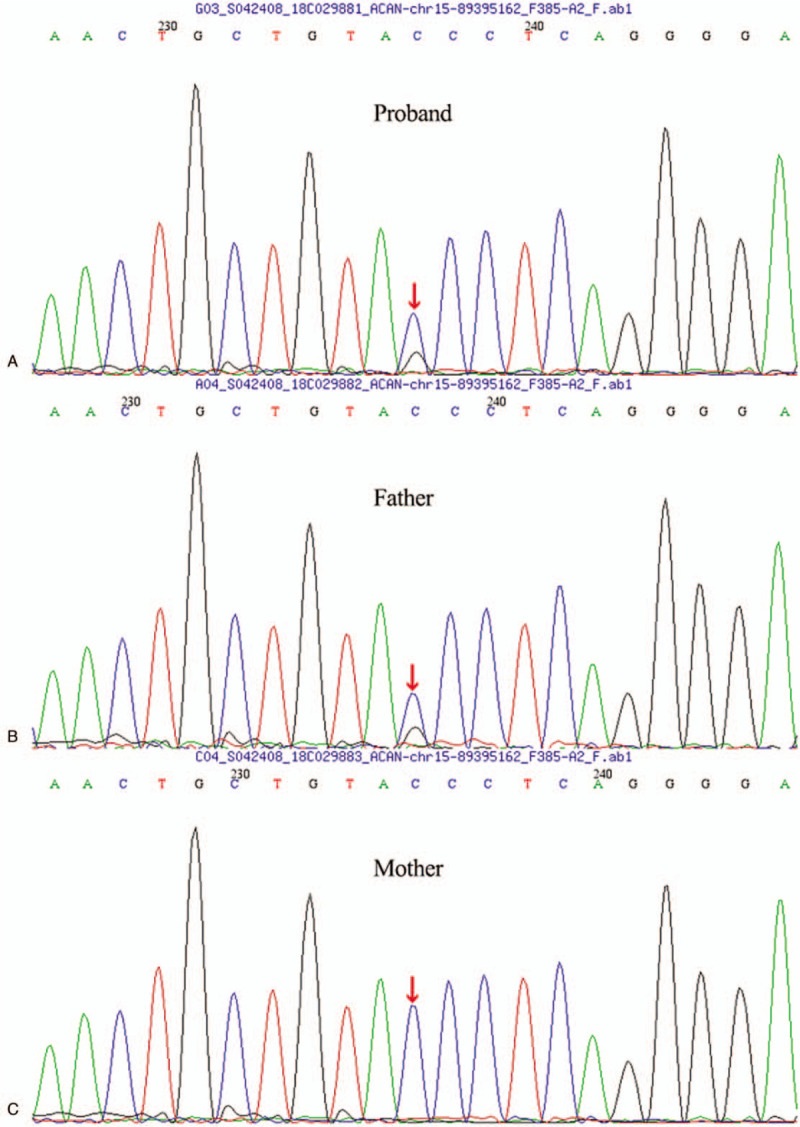
Sanger traces for PCR products of the patient and his parents. A Sanger traces for PCR products of the patient indicated a heterozygous mutation. The nucleotide at position 2164 changed from cytosine to guanine, resulting in mutation at amino acid 722 of the encoded protein from proline to alanine, c.2164C>G (p.P722A) b,c Sanger traces for PCR of his parentsb(b for the proband's father, carry this same heterozygous mutation c. 2164C>G (p.P722A), c for the proband's mother,did not carry this mutation). PCR = polymerase chain reaction.

A 5-year-4-month-old full-term twin test-tube girl delivered by cesarean section was admitted to the Outpatient Department of our hospital due to short stature on September 25, 2018. The child also has a sibling brother, and there is no consanguineous marriage in her parents. The child has a birth weight of 2.3 kg (-3SDS) and a birth length of 46 cm (-2.4SDS). Her growth rate is slower than that of her peers, yet with unknown specific growth rate. The child had a body weight of 99.4 cm (-3.13SDS) on admission and a bone age (BA) of 5 years old, lagging behind the actual age. Her father's height is 153 cm (-3.3SDS), her mother's height is 169 cm (+1.58SDS), and the height of twin brother is 110.5 cm (-0.93SDS). Physical examination: No deformity in appearance, as well as no abnormality in organ examination and asexual development. Further laboratory examination showed no abnormality in routine blood and urine tests, liver function, tandem mass spectrometry in blood and urine, chromosomal karyotypes, thyroid function, ACTH and cortisol. There was no abnormality in gynecological ultrasound, X-ray of spineand pituitary MRI. The peak value of GH provocative test was 9.53ng/mL via oral administration of clonidine, which was 8.34ng/mL via intravenous injection of insulin, and the insulin-like growth factor-1 (IGF-1) was detected to be 267.8ug/L. According to the test results, the pediatric patient was provided with recombinant human GH (rhGH; 0.1iu/kg.d) for 6 months. The growth rate of the child was 8 cm/year, and the body height was 103.8 cm (-2.78SDS). However, subsequent physical examination found that the girl had bilateral breast development 6 months later. Physical examination showed that the breast of the pediatric patient was in stage B2, with induration but no tenderness, or development of pubic hair and axillary hair. The peak value of luteinizing hormone was tested to be 5.75mLU/mL after gonadotropin releasing hormone stimulation test for 30 min using Triptorelin (Diphereline); LH/FSH was greater than 0.6; and the BA was equivalent to 6 years old at the age of 5 years and 10 months. Accordingly, rhGH was increased to 0.15iu/kg.d for treatment in combination with gonadotropin releasing hormone analoges (GnRHa) for 1.88 mg/4 weeks. the pediatric patient was followed up every half a year for a total of 1 year. The growth rate was 8.1 cm/year and the height was111.9cm(-2.08SDS) at the age of 6 years and 10 months (Fig. 1), with the retraction of the secondary sexual character of breast development. Besides, re-examination of estrogen and luteinizing hormone revealed decrease to the prepuberal level, with no significant increase in BA (Fig. 2). The target high-throughput sequencing was carried out by using a Capture Kit containing 4,437 known pathogenic genes (Mygenostics, Beijing, China). The results showed that there was a missense mutation in exon 11 of ACAN gene (NM-0132). Specifically, the nucleotide at position 2,164 changed from cytosine to guanine, resulting in the mutation at amino acid 722 of the encoded protein from proline to alanine, i.e., c.2164C>G (p.P722A), which was a heterozygous mutation. Further family validation analysis showed that her father was a heterozygous carrier for this mutation, while no mutation was found in her mother (Fig. 3). There is no report of this site in Human Gene Mutation Database (HGMD). The mutation was predicted to be harmful by using REVEL, a bioinformatics protein function prediction software. The present case report has been approved by the Review Committee of Qingdao Women and Children's Hospital, and the study has obtained the informed consent of the parents of the pediatric patient.

## 3 Discussion

Increasingly more genes related to short stature have been found on the basis of the development of the etiology of short stature and gene sequencing techniques. ACAN is 1 of the discovered genes, which is located on chromosome 15q26 and consists of 19 exons, ranging in size from 77 bp to 4224 bp.^[3]^

To date, 64 mutations in ACAN gene have been registered in HGMD, including 22 nonsense mutations, 22 missense mutations, 2 shear mutations, 12 small deletion mutations, 3 insertion mutations, 2 insertion and deletion mutations, 1 large deletion mutation, 2 repeat mutations and 1 gene rearrangement. Among them, 87.9% of the mutations have the characteristic of inheritance, and only a few short stature children with ACAN gene mutation are sporadic.^[4]^ The case reported in this study was a small for gestational age (SGA) child. Thehigh-throughput sequencing showed that there was a missense mutation in exon 11 of ACAN gene (NM-0132). The nucleotide at position 2,164 changed from cytosine to guanine, resulting in mutation at amino acid 722 of the encoded protein from proline to alanine, ie, c.2164C>G (p.P722A), which was a heterozygous mutation. Her father was found to be a heterozygous carrier for this mutation. It suggests that short stature caused by ACAN and gene mutations shall be highly suspected when the short stature children have obvious family genetic history. Simultaneously, literature review found no similar mutation.

Mutations in ACAN gene can lead to a broad phenotype spectrum of nonfatal dysplasia of bone, including spondyloepiphyseal dysplasia, spondyloepiphyseal dysplasia Kimberley type, familial osteochondritis dissecans, and various idiopathic dwarf phenotypes.^[6,7]^ ACAN encodes proteoglycan and extracellular matrix, both of which are key components for the structure and function of growth plate cartilage and other cartilage tissues,^[8]^ which may be responsible for the pathogenesis of osteochondritis in some patients. The antiangiogenic function of proteoglycan aggregation has been proposed at the same time.^[9]^ In this regard, ACAN gene mutation may cause premature or increased invasion of growth plate by blood vessels and osteoblasts, leading to ossification of growth cartilage, advanced BA, early epiphyseal fusion, and premature growth stop. However, it still remains unclear concerning the exact mechanism of different mutations in the ACAN gene leading to a series of phenotypes. It can be confirmed so far is that the affected individuals were short and stopped growing at an early stage in all published studies. It is consistent with the report of this case.

In this case report, the child has a short stature only and SGA at birth. In the International Small for Gestational Age Advisory Board Consensus Development Conference Statement in 2001, it was proposed that a child aged between 2 to 3 years old who is SGA, is not likely to catch up and has persistent short statureshould be referred to a pediatrician who has expertise in endocrinology for assessment and guidance.^[5]^ Manouk et al reported that the positive rate was 13.8% during a test of 29 SGAs with short stature and advanced BA. With respect to the above, there is a need to consider ACAN gene mutation in SGA patients with normal or advanced BA, those with no significant growth catch-up in the later period, and those with family history. Early diagnosis and treatment, especially for this case with precocious puberty, will provide additional therapeutic space and time before epiphysis healing.

In a study involving 20 families with variant ACAN, 62.5% of the children showed advanced BA of different degrees.^[11]^ Hence, advanced BA had been recognized as 1 of the typical characteristics of short stature caused by ACAN gene mutation. However, recent studies have reported that backward or normal BA can also be found in a small number of patients as well.^[10]^ In this case, the pediatric patient showed lagged BA at the beginning, but combined CPP within half a year of follow-up, accompanied by accelerated BA by 1 year (Fig. 2). It not only confirms that BA is not the feature for differential diagnosis of ACAN gene mutation, but also suggests that the feature of BA may have no obvious correlation with the genotype.

Furthermore, in a cohort study carried out by Quintos et al, 12 out of 20 families^[7,8,9,12,13]^ had not only short stature, but also premature onset osteoarthritis, with knee pain as the most frequent symptom. In another cohort study by Gkourogianni, affected individuals from several families also had early-onset disc disease,^[14]^ in which severe osteoarthritis was found in the second and fourth decades of life. Notably, the time node of early osteoarthritis and intervertebral disc disease has not been reached in our case report. Thus, for our case reported here, there is a need for active follow-up and timely treatment in the case of such situation to delay the occurrence of disability and to improve survival treatment.

In terms of treatment, the height of the child was 99.4 cm (-3.13SDS) on admission, which was improved to 103.8 cm (-2.78SDS) after rhGH therapy (0.1–0.15iu/kg), with a growth rate of 8 cm/year. Concerning the curative effect of GH alone, it has been reported that the final height (-2.5 SD) of 5 adults treated with GH was slightly better than that of 65 adults without GH treatment (-3.0 SD).^[7]^ However, the special situation was that the child had breast development after 6 months of treatment, which was diagnosed as idiopathic CPP after gonadotropin releasing hormone stimulation test using Triptorelin (Diphereline). To this date, there is no international report of ACAN mutation with precocious puberty, and our study reported for the first time. It is speculated that in children with ACAN gene mutation, patients with advanced BA before puberty tend to have height growth stagnation after puberty. In the families reported in prior research, the predicted adult height of the elder brother with combined therapy of GH and GnRHa was 13 cm higher than that of the younger brother with GH treatment alone.^[15]^ Considering the presence of precocious puberty in this case, there was no obvious increase in BA in the follow-up for half a year after GnRHa treatment. However, it is still necessary to consider that some of the children who received combined therapy of GH and GnRHa have not reached adult height, and corresponding clinical effect needs further observation and evaluation. Simultaneously, it requires further clinical studies with larger sample size to confirm the time nodes of add-on therapy of GnRHa.

At present, increasingly more single gene disorders are diagnosed by high-throughput sequencing technology. For children with ISS, especially those with family history, or SGA only, and unsatisfactory growth catch-up, gene diagnosis technology shall be applied in time for early diagnosis to strive for more growth time and improve the growth quality of children. Meanwhile, it is worth noting that GH may promote height development by stimulating IGF-1 production and chondrocyte differentiation. Therefore, it is unlikely that the defects of proteoglycan aggregation can be repaired by GH alone. Molecular biology research may be required in future to restore normal proteoglycan aggregation to improve the treatment of children with ACAN gene mutation.

## Author contributions

**Conceptualization**: YuanyuanWang, JuanGe, JianyinMa, Lingyan Qiao, Tang Li.

**Data curation**: YuanyuanWang, JuanGe.

**Formal analysis**: YuanyuanWang, JuanGe, JianyinMa, Lingyan Qiao, Tang Li.

**Fundingacquisition**: YuanyuanWang, JuanGe, JianyinMa, Lingyan Qiao, Tang Li.

**Investigation**: YuanyuanWang, JuanGe, JianyinMa, Lingyan Qiao, Tang Li.

**Methodology**: YuanyuanWang, JuanGe, JianyinMa, Lingyan Qiao, Tang Lii.

**Project administration**: YuanyuanWang, Tang Li.

**Resources**: YuanyuanWang, JuanGe, JianyinMa, Tang Li.

**Software**: YuanyuanWang, JuanGe, JianyinMa, Tang Li.

**Supervision**: YuanyuanWang, JuanGe.

**Validation**: YuanyuanWang, JuanGe, JianyinMa.

**Visualization**: YuanyuanWang, JuanGe, Lingyan Qiao.

**Writing – original draft**: YuanyuanWang, JuanGe, Lingyan Qiao.

**Writing – review & editing**: YuanyuanWang, JuanGe.
